# Wide-thread implant macrogeometry and immediate implant placement: A systematic review of primary stability, marginal bone loss, and survival outcomes

**DOI:** 10.4317/jced.63890

**Published:** 2026-03-30

**Authors:** Rubén Jiménez Hernández

**Affiliations:** 1 DDS. Adjunct Clinical Assistant Professor, Department of Prosthodontics, Faculty of Health Sciences, Catholic University of Murcia (UCAM), Murcia, Spain

## Abstract

**Background:**

Wide-thread dental implants have been proposed to enhance primary stability and marginal bone preservation in immediate implant placement protocols, particularly in low-density bone. This systematic review aimed to evaluate the influence of wide-thread implant macrogeometry on primary stability, marginal bone loss, and survival outcomes in immediate implant placement.

**Material and Methods:**

A systematic electronic search was performed in PubMed (MEDLINE), complemented by manual screening of reference lists and forward and backward citation tracking, to identify studies published between January 2007 and August 2025. Clinical studies, in vitro experiments, finite element analyses, and reviews assessing the effect of implant macrogeometry, especially wide-thread configurations, on insertion torque, implant stability quotient (ISQ), marginal bone loss (MBL), and survival in immediate placement were included. Studies without biomechanical relevance, non-English publications, case reports, and those lacking quantitative stability or bone data were excluded.

**Results:**

A total of 36 records were initially identified, and 25 studies met the inclusion criteria. Increased thread depth, wider or more aggressive threads, and tapered implant bodies generally improved insertion torque and ISQ values, particularly in D3-D4 bone and post-extraction sockets. Clinical investigations reported high survival rates for immediately placed implants with optimized macrogeometry, while MBL typically remained within clinically acceptable limits when adequate primary stability and appropriate loading protocols were achieved. However, small sample sizes, short follow-up periods, heterogeneity among implant systems, and non-standardized outcome reporting limited the possibility of robust quantitative comparisons.

**Conclusions:**

Wide-thread implant macrogeometry appears to enhance primary stability in immediate placement and supports favorable short- to medium-term survival and marginal bone behavior when combined with careful case selection and controlled loading. Further well-designed prospective randomized studies with long-term follow-up are needed to determine whether these biomechanical advantages translate into superior long-term clinical outcomes.

## Introduction

Over the past two decades, oral implantology has evolved substantially, driven by the aim of reducing treatment times and enhancing clinical predictability-even in demanding scenarios such as immediate post-extraction placement. This approach, in which the implant is inserted at the time of tooth removal, offers clear advantages, including fewer surgical interventions and preservation of alveolar bone volume. However, it also poses considerable biomechanical challenges, particularly achieving adequate primary stability in sites with low bone density or compromised bony walls. Primary stability-defined as the absence of implant mobility at placement-is considered one of the main predictors of success under immediate loading protocols. It is directly influenced by the implant's macrogeometry, including variables such as body shape, thread profile, and thread depth. Within this context, wide-thread implants have gained particular interest. This design increases the contact between the implant surface and bone, supporting higher insertion torque and a more favorable distribution of forces within the peri-implant bone, which is especially advantageous in D3-D4 bone. It has also been proposed that such geometry can compensate for limited corticalization or low trabecular density, increasing the initial stiffness of the implant-bone complex and facilitating early or immediate loading. Despite these potential benefits, the literature on the clinical performance of wide-thread implants under immediate placement exhibits heterogeneity in study design, follow-up duration, and success criteria. A critical appraisal of the available evidence is therefore essential to establish robust conclusions regarding the clinical applicability of wide-thread implants. Although some reviews have addressed implant macrogeometry in general, there are limited studies specifically focused on wide-thread implants under immediate placement protocols. This review aims to critically integrate and evaluate both biomechanical and clinical evidence to clarify their potential advantages and limitations in these demanding scenarios. Although research on implant macrogeometry has increased, the available evidence remains fragmented across experimental, numerical, and clinical domains, with inconsistent outcome reporting and limited integration between biomechanical findings and clinical performance under immediate placement protocols. Previous reviews have generally addressed implant design in broad terms or have focused on isolated parameters, without providing a structured synthesis specifically centered on wide-thread macrogeometry as a design variable in immediate implant placement. Consequently, there is a lack of consolidated evidence clarifying how biomechanical advantages observed in experimental and finite element models translate into clinically relevant outcomes such as primary stability, marginal bone loss, and implant survival. Addressing this gap is essential to support evidence-based decision-making in demanding immediate placement scenarios. Therefore, this systematic review aims to analyze the impact of wide-thread macrogeometry on key clinical parameters-namely insertion torque, primary stability measured by the Implant Stability Quotient (ISQ), marginal bone loss (MBL), and implant survival-focusing specifically on immediate placement protocols. To this end, we included clinical, biomechanical, in vitro, and finite element analysis (FEA) studies published between 2007 and 2025, rigorously selected and screened by the author according to predefined eligibility criteria.

## Material and Methods

**Figure 1:**
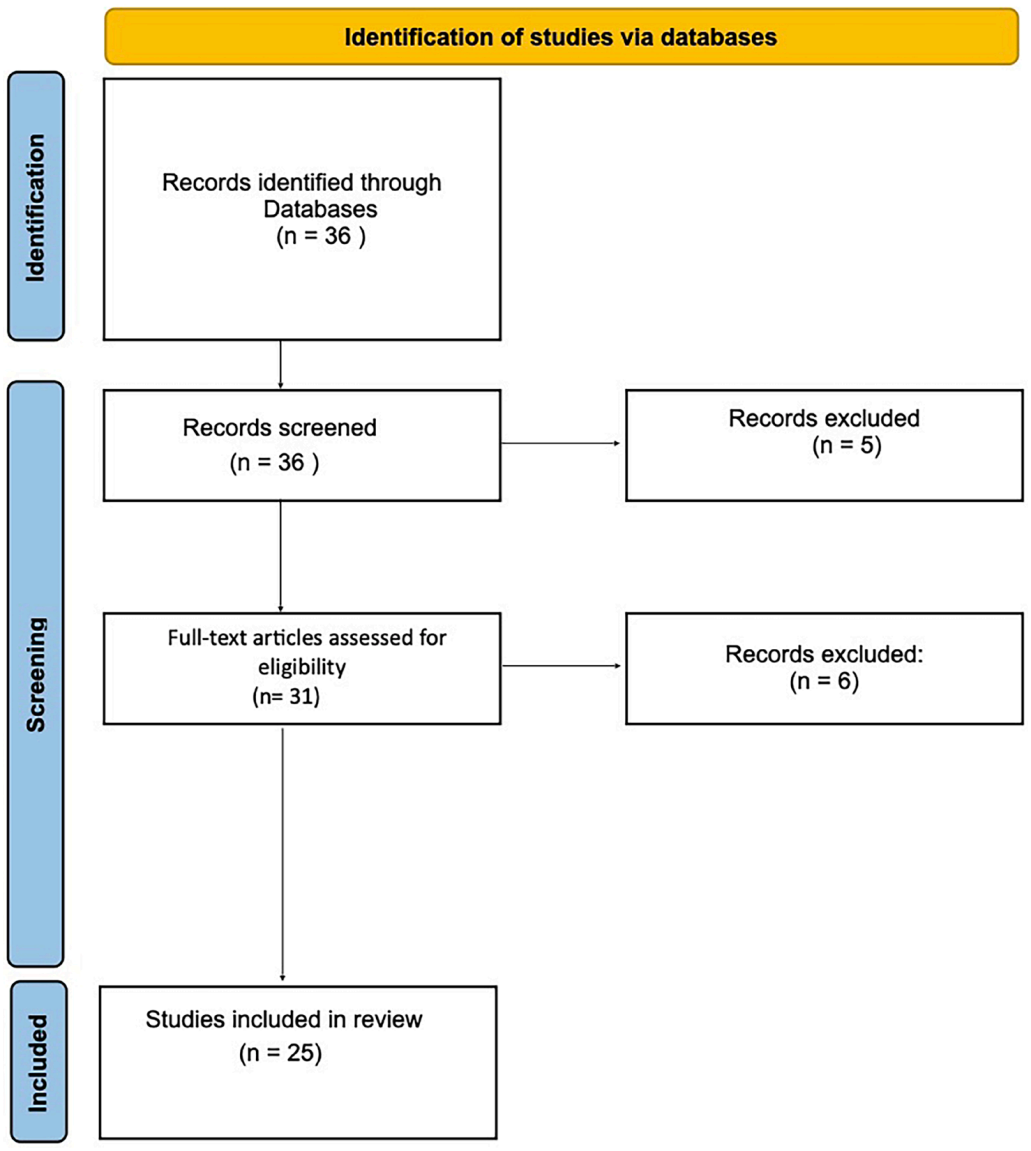
PRISMA 2020 flow diagram.

1. Study design and research question This study was conducted as a systematic review of the literature, following PRISMA 2020 principles for identification, screening, and reporting of eligible studies. The review addressed the following research question: In immediate implant placement protocols, how does wide-thread implant macrogeometry influence primary stability (insertion torque and implant stability quotient), marginal bone loss, and implant survival compared with conventional thread designs or non-specified macrogeometries? Clinical studies, in vitro experiments, finite element analyses (FEA), and relevant reviews published between 2007 and 2025 were considered to provide a comprehensive evaluation of biomechanical and clinical outcomes. 2. Protocol and registration A formal review protocol was developed a priori, defining the research question, eligibility criteria, search strategy, and data extraction plan; however, the protocol was not registered in PROSPERO or other public databases. The review adhered to PRISMA-based transparency, including a flow diagram to document study identification, screening, eligibility assessment, and final inclusion. 3. Search strategy An electronic literature search was performed in PubMed (MEDLINE) between May 1 and August 31, 2025, covering publications from January 2007 to August 2025. The final search was completed on August 31, 2025. PubMed was selected as the primary database because of its comprehensive coverage of biomedical and dental literature and its widespread use in systematic reviews in the health sciences. The search strategy combined free-text keywords and Boolean operators related to implant macrogeometry and immediate placement. A representative search string applied in PubMed was: ("dental implant") AND ("implant macrogeometry" OR "thread design" OR "wide-thread implant") AND ("immediate implant placement" OR "primary stability" OR "insertion torque" OR "implant stability quotient"). To reduce the risk of missing relevant studies, manual screening of reference lists and forward and backward citation tracking of eligible articles were also performed. No restrictions on study design were applied in order to capture clinical studies, in vitro experiments, finite element analyses (FEA), and relevant reviews addressing the biomechanical and clinical implications of implant thread macrogeometry. 4. Eligibility criteria Studies were eligible if they directly or indirectly evaluated the influence of implant macrogeometry in the context of immediate implant placement. Inclusion criteria comprised investigations focusing on thread design, thread pitch, depth, diameter, or other macrogeometric features with reported outcomes for insertion torque, primary stability, ISQ, MBL, or survival. Exclusion criteria were: (1) articles unrelated to implant macrogeometry; (2) studies not involving immediate placement or lacking biomechanical relevance; (3) case reports; (4) publications not written in English; and (5) studies without extractable quantitative data on stability or bone-related outcomes. 5. Study selection and PRISMA flow A total of 36 records were initially identified through database searching and additional manual screening. Titles and abstracts were screened according to predefined eligibility criteria, and potentially eligible articles underwent full-text assessment. 31 full-text articles were assessed, of which 25 met the inclusion criteria and were incorporated into the qualitative synthesis. The study selection process is detailed in a PRISMA 2020 flow diagram (Fig. 1).

1

6. Data extraction and outcomes Data extraction was performed manually using a predefined template. When available, the following variables were collected: authors and year; study design (clinical, in vitro, FEA, review); implant macrogeometric characteristics (thread depth, pitch, shape, diameter, taper); sample size (patients and/or implants); anatomical location; insertion torque (IT); ISQ values; marginal bone loss (MBL); follow-up duration; and survival or success rates. Screening and data extraction were performed by a single reviewer using predefined criteria. To minimize potential selection bias, all included studies were reassessed during the data extraction phase to confirm eligibility and consistency of the extracted information. Repeated full-text assessment was also performed to ensure internal consistency of the extracted data. 7. Data synthesis Given the substantial heterogeneity in methodologies (FEA, in vitro, retrospective, and prospective designs), outcome measures (IT, ISQ, MBL, survival), implant systems, and follow-up durations, a quantitative meta-analysis was not feasible. Therefore, data were synthesized qualitatively and organized into thematic domains: (1) implant macrogeometry; (2) primary stability; (3) survival of immediately placed implants; (4) marginal bone loss and medium- to long-term behavior; and (5) strengths and limitations of the available evidence. This approach allowed structured integration of biomechanical and clinical findings while acknowledging inter-study variability. Because the included literature comprised heterogeneous study designs (clinical studies, in vitro experiments, finite element analyses, and reviews), the interpretation of evidence followed a hierarchical approach in which clinical studies were prioritized over experimental and simulation-based evidence, which was considered supportive of biomechanical interpretation. 8. Risk of bias assessment Risk of bias assessment was performed according to the study design. The randomized clinical trial was evaluated using the Cochrane Risk of Bias 2 (RoB 2) tool. Non-randomized clinical studies were assessed using the Newcastle-Ottawa Scale (NOS), which evaluates selection, comparability, and outcome domains. NOS scores were interpreted according to commonly used thresholds in observational studies (7-9 low risk, 5-6 moderate risk, &lt;5 high risk). The risk-of-bias assessment was conducted by the reviewer based on the methodological information reported in each study, following the criteria established by the RoB 2 and Newcastle-Ottawa Scale instruments. In vitro studies, finite element analyses, and narrative reviews were not subjected to formal clinical risk-of-bias tools, as these instruments are not designed for experimental or simulation-based evidence. These studies were interpreted as mechanistic or contextual support and considered separately from clinical evidence. Overall, most included clinical studies were judged to have a moderate risk of bias, mainly due to limited sample sizes, heterogeneity in follow-up protocols, and the predominance of retrospective and non-randomized study designs. Most observational studies achieved Newcastle-Ottawa Scale scores between 5 and 6 points, corresponding to moderate methodological quality and limited control of confounding variables, whereas the randomized clinical trial showed a low risk of bias. The results of the risk-of-bias assessment are summarized in (Supplement 1) http://www.medicinaoral.com/medoralfree01/aop/jced_63890_s01

## Implant Macrogeometry and Biomechanical Principles

1. Definition of Wide-Thread Design The term wide-thread refers to a thread design characterized by a larger thread pitch, increased thread depth, and a more pronounced thread width. These features enhance the contact surface between the implant and the surrounding bone, thereby promoting compression of the osteotomy site during insertion. Thread pitch refers to the distance between consecutive threads; while reducing the pitch may increase the total threaded surface, it can compromise implant advancement in dense bone. Thread depth, in turn, determines the implant's ability to engage cortical or cancellous bone, whereas thread width influences mechanical strength and the cutting efficiency of the implant. In an experimental study, Cucinelli (1) compared implants with different thread depths in D3-type bone, showing that deeper thread designs achieved more efficient and stable insertion. In line with this, Ormianer (2) provided strong clinical evidence demonstrating that implants with wide and deep threads exhibited reduced marginal bone loss (MBL) at long-term follow-up compared to relatively conventional designs. This highlights the role of thread macrogeometry as a determinant not only of primary stability but also of peri-implant bone preservation over time. In a review, Huang (3) emphasized that thread geometry-including pitch, depth, and shape-plays a decisive role in both primary stability and stress distribution within the surrounding bone. The author also noted that different thread configurations, such as square, V-shaped, buttress, and reverse buttress, present specific biomechanical properties tailored to distinct clinical scenarios. Complementary evidence comes from earlier reviews by Abuhussein (4) and Ryu (5), which explored the influence of these parameters on thread biomechanics. Abuhussein (4) reported that thread geometry is critical for stress distribution around the implant and that wider and deeper threads improve primary bone contact and mechanical retention in low-density bone, thereby enhancing functional load response. Ryu (5) reinforced this evidence through a literature review, noting that a square thread profile may provide superior primary stability under immediate loading conditions. Moreover, the author pointed out that a smaller thread pitch increases bone-implant contact, improving initial fixation, while thread depth is essential for dissipating peak stresses within the bone. Together, these findings emphasize that the optimal combination of thread shape, pitch, and depth can enhance both primary stability and load distribution in immediate placement protocols. Consistent with these observations, the work of Ariani Ayub (6) also supports the role of wide and deep threads, demonstrating that more aggressive macrogeometric configurations, such as double- or triple-thread designs, enhance implant engagement in low-density bone and represent a recommended strategy for compromised bone quality conditions. Overall, the available evidence supports the use of wide and deep threads as an effective approach to optimize primary implant fixation while preserving the integrity of the surrounding bone microstructure. 2. Biomechanical Principles From a biomechanical perspective, the wide-thread design plays a significant role in optimizing force distribution during the functional loading phase of the implant. Increasing the contact surface between the implant and the bone not only improves initial anchorage but also reduces stress concentration in critical areas of the cortical bone. In a three-dimensional finite element analysis (FEA), Alqahtani AR (7) observed that implant diameter directly influences stress distribution, strain, and micromovements, with larger-diameter implants exhibiting more favorable behavior compared to narrower ones. Moreover, vertical loads produced lower stress levels than oblique loads. Regarding thread macrogeometry, the square thread design demonstrated superior biomechanical performance by distributing stresses more homogeneously within the surrounding bone compared with alternative configurations such as triangular or buttress threads. These findings highlight the relevance of both implant diameter and thread morphology in the biomechanical behavior of short implants, particularly in scenarios with limited bone height. In addition, the tapered apical design may also be associated with significant improvements in initial stability by promoting the "self-tapping" effect of the implant within cancellous bone. This is reflected in the clinical data reported by Coppedê (8), who evaluated TRI Bioneck RP implants (Derig®) featuring a tapered apical body and cervical microthreads, placed under All-on-Four protocols with high insertion torque values (75 Ncm). After 24 months of follow-up, no biomechanical complications attributable to the elevated torque were observed, confirming that high torque values can be clinically safe in immediate and early loading protocols. Herekar (9), through FEA, investigated how microthread configuration influences stress transfer to bone, comparing designs with multiple microthreads (four-fold microthread) versus a single-pitch microthread. The results showed that the multiple microthread model favored better stress distribution in trabecular bone and within the implant itself, whereas the single-pitch design reduced stress concentration in cortical bone, albeit at the cost of greater loads on the implant. However, the authors concluded that the single-pitch design was generally more favorable and applicable across different bone configurations, conclusions that were not fully consistent with their own reported results. This discrepancy underscores the need for cautious interpretation and highlights the importance of clinical studies to validate the real-world applicability of such simulations. The review by Ryu (5) supports this perspective, emphasizing that implant macrodesign factors are decisive in biomechanical behavior, influencing stress distribution in the crestal cervical bone region and, ultimately, primary stability in cancellous bone. The author highlights that thread shape, along with depth and pitch, plays a critical role in stress dissipation and effective transmission of functional loads during early osseointegration, with square thread profiles and reduced thread pitch emerging as the most favorable configurations in this context. Taken together, the evidence suggests that wide-thread macrogeometry improves immediate mechanical parameters (torque and ISQ) and contributes positively to load biomechanics, bone stability, and implant survival. Its use is particularly indicated in low-density bone, post-extraction, or immediate loading scenarios, where primary stability requirements are more demanding. Nevertheless, while experimental and simulation-based evidence supports these advantages, well-designed clinical trials with long-term follow-ups are still required to validate their impact on implant survival and the maintenance of peri-implant bone stability. The available literature on implant macrogeometry includes heterogeneous types of evidence, ranging from experimental and finite element analyses to clinical investigations. While biomechanical studies provide mechanistic insights into implant-bone interactions, clinical studies remain the primary source of evidence for evaluating real-world outcomes such as stability, survival, and marginal bone loss.

## Primary stability: Effect of wide-thread design on insertion torque and ISQ

1. Insertion Torque Insertion torque (IT) reflects the bone's resistance to implant advancement during placement, and its numerical value serves as a reference for determining the feasibility of immediate loading protocols. Baldi (10) reported insertion torques ranging from 18.8 ± 6 to 68.2 ± 12.1 Ncm in patients receiving tapered AnyRidge implants (Megagen®), 4 mm in diameter and 10 mm in length, characterized by wide, deep, self-tapping threads. A significant positive correlation was also observed between insertion torque and ISQ values, confirming that wide-thread macrogeometry is a decisive factor in achieving the high levels of primary stability required for predictable clinical outcomes. Shiigai (11) observed in in vitro tests that implants with triple-thread tapered designs achieved mean IT values of 49 Ncm, slightly higher than those of straight implants. In patients, triple-thread implants recorded an average IT of 43.2 Ncm, consistent with the optimal ranges for primary stability. In the in vitro study by Cucinelli (1), which specifically focused on wide-thread implants, it was demonstrated that increasing thread depth significantly increased the torque required to insert implants in D3 low-density bone, thereby improving primary stability. A positive correlation was observed between IT and ISQ. Specifically, AnyRidge (Megagen®) implants with deeper threads reached a mean IT of 54.03 ± 8.99 Ncm and ISQ of 70.13 ± 1.12, compared with 25.12 ± 2.96 Ncm and ISQ 65.58 ± 1.37 for shallower-thread implants. Despite its experimental nature, this finding is particularly relevant, as it was based on a single wide-thread implant model to isolate the biomechanical behavior of different thread depths. These results are clinically complemented by Baldi's (10) patient-based study using the same implant model. Similarly, Ariani Ayub (6) conducted an in vitro study with 36 implants from three systems with distinct macrogeometric thread configurations: Straumann® BLT, Zimmer® TSV, and Dentium® Superline. Implants were placed in simulated bone blocks with densities equivalent to D3 and D4, and insertion torque (IT) and primary stability were assessed by ISQ. In D3 bone, Zimmer TSV showed the highest IT (30.21 ± 1.38 Ncm), while Dentium Superline recorded the highest ISQ (63.29 ± 0.54). In D4 bone, Zimmer TSV again reached the highest IT (18.07 ± 1.71 Ncm), whereas Dentium Superline maintained the highest ISQ (58.46 ± 0.78). Based on these findings, the author emphasized that tapered implants with deeper and more aggressive threads, including double- or triple-thread configurations, are especially recommended for patients with compromised bone quality, as they provide better engagement and superior primary stability-critical factors in immediate or early loading protocols. Further evidence comes from Yamaguchi (12), who performed another in vitro study using artificial bone to evaluate three thread configurations: a single-thread implant with a 1.2 mm pitch (control); a double-thread implant with a 0.6 mm pitch and 1.2 mm lead; and a single-thread implant with a 0.6 mm pitch. Primary stability and ISQ values were analyzed. Results indicated that both modified designs significantly improved primary stability compared to the control. The author concluded that primary stability may be positively influenced, especially in low-density bone, by extending thread length and reducing pitch/lead. These findings reinforce the principle that increasing the bone-implant contact surface enhances primary stability-consistent with the effective surface area achieved by wide-thread designs. In the clinical setting, Menini (13) conducted a prospective study on 14 patients, comparing implants with standard threads (SY) and wide threads (SL) of the Syra model (Sweden &amp; Martina®). Each patient received at least one implant of each type, for a total of 38 implants (19 SL and 19 SY). After 12 months of follow-up, mean insertion torque values were higher in wide-thread SL implants (48.42 Ncm) compared to standard-thread SY implants (43.42 Ncm), though differences did not reach statistical significance. Nevertheless, this clinical evidence confirms that wide-thread macrogeometry can enhance primary stability even when all other design and surface parameters are held constant. This is one of the few clinical studies directly comparing two thread variants of the same implant platform, conferring strong methodological value by demonstrating, in controlled clinical conditions, the specific contribution of wide threads to initial implant fixation. In his review, Huang (3) noted that primary stability depends on both macro- and micro-design, surgical technique, and bone quality; and that insertion torque (IT) and ISQ remain the most commonly used metrics for its evaluation. He also highlighted the need to control micromovements ( &lt; 0.1 mm) during immediate loading protocols to ensure osseointegration. Lozano-Carrascal (14) reported significant differences in primary stability according to anatomical location: in the mandible, mean torque was 46.67 ± 6.85 Ncm for tapered implants versus 35.77 ± 6.72 Ncm for cylindrical ones (p = 0.01), whereas in the maxilla, values were 41.5 ± 6.26 Ncm and 39.17 ± 6.34 Ncm, respectively (p &gt; 0.05). Ryu (5) pointed out that tapered designs with aggressive threads may enhance primary stability in compromised bone and are more suitable for immediate loading protocols, although available evidence is primarily based on experimental studies and reviews, without conclusive clinical data on insertion torque. The systematic review by Oliveira (15) reinforced the importance of primary stability in immediate loading protocols. Including six clinical studies, the review concluded that there is insufficient evidence to assert that an insertion torque 30 Ncm alone guarantees implant survival; however, success rates above 95% were consistently observed when a minimum IT of 30 Ncm was achieved. In this context, wide-thread designs, due to their increased bone-implant contact surface, may contribute to achieving the torque levels required to enhance primary stability and thereby improve treatment predictability. 2. Resonance Frequency Analysis (RFA) The implant stability quotient (ISQ), obtained through resonance frequency analysis (RFA), provides a quantitative and noninvasive assessment of implant stability. ISQ values 70 are generally considered desirable in immediate loading protocols. In the clinical study by Baldi (10), which involved the placement of wide-thread implants in patients and was previously discussed in section 3.1, ISQ values ranged between 71.8 ± 6.6 and 78 ± 6.4, directly correlating with insertion torque. Shiigai (11) reported, through in vitro testing, that triple-thread tapered implants achieved ISQ values of 70, significantly higher than those of straight implants. In patients, triple-thread implants recorded a mean ISQ of 71.8 at placement, which remained stable throughout the 12-week follow-up. In the in vitro study by Cucinelli (1), beyond its influence on insertion torque, increased thread depth was also associated with higher ISQ values. Specifically, implants with deeper threads reached a mean ISQ of 70.13 ± 1.12 compared with 65.58 ± 1.37 in shallower-thread implants, reinforcing the role of RFA as a reliable indicator of primary stability. Additionally, Lozano-Carrascal (14) assessed ISQ values across different designs and anatomical locations, finding significant differences. In the mandible, tapered implants yielded a mean ISQ of 71.67 ± 5.16 compared to 57.15 ± 4.83 in cylindrical implants (p = 0.01), whereas in the maxilla, the values were 67.2 ± 4.42 and 49.17 ± 15.30, respectively (p = 0.01). These findings reinforce the positive association between insertion torque and ISQ observed in the same study, confirming that tapered geometry provides biomechanical advantages, particularly in low-density bone. Clinically, Coppedê (8) reported follow-up data on TRI Bioneck® RP implants (Derig®) placed under All-on-Four protocols in 11 patients (43 implants). In addition to elevated insertion torques (75 Ncm), consistent ISQ values were maintained over 24 months without decreases that would compromise osseointegration. These results suggest that both insertion torque and stability measured by RFA can remain within clinically safe ranges under immediate protocols, reinforcing the combined utility of these metrics for monitoring primary stability over time. Collectively, these findings establish ISQ as a reliable tool for monitoring primary stability and its evolution over time, both in experimental studies and in immediate clinical protocols. Nevertheless, although the positive correlation between insertion torque and ISQ is consistent, its predictive value for long-term clinical success still requires validation through controlled trials and extended follow-ups. Table 1 presents insertion torque and ISQ values according to thread design from experimental and clinical studies.

Table 1

## Survival Rates of Wide-Thread Implants in Immediate Placement

1. Short- and Long-Term Clinical Outcomes Survival rates constitute the main clinical indicator for assessing the reliability of immediately placed implants and, consequently, the viability and predictability of this protocol. This section examines how implant macrogeometry, and in particular thread design, influences the survival rate of implants placed under immediate protocols. Wipawin (16) conducted a 3-5 year clinical follow-up in 19 patients with 25 Straumann® bone-level tapered SLActive implants placed immediately in posterior regions. The survival rate was 96%, with no failures during the first year, and only two prosthetic complications were reported during maintenance: loss of proximal contact and screw loosening. These results support the clinical viability of implants with optimized designs to ensure stability under immediate loading protocols. Meijer (17) published a prospective study involving 15 NobelActive implants (Nobel Biocare®) with deep-thread macrogeometry, 4.3 mm in diameter and 8.5-10 mm in length, placed immediately in molar sites. After one year of evaluation and follow-up, the overall survival rate, including prosthetic restorations, was 73.3% due to several early failures. Nevertheless, implants that remained in function showed stable osseointegration without pathological bone loss or prosthetic complications. Despite its limited sample size and short follow-up, this study provides evidence of the viability of immediate protocols in posterior sites when deep-thread macrogeometries are employed. Additionally, Aung (18) analyzed the long-term survival of bone-level tapered self-tapping implants placed under immediate protocols. A total of 49 Luna® implants were inserted in 34 patients, 23 in the mandible and 26 in the maxilla. The mean follow-up period was 7.43 years, ranging from 5 to 14 years. After a 5-year retrospective evaluation, the cumulative survival rate reached 93.88%. The author concluded that tapered, self-tapping implants provide predictable long-term results when used in immediate placement, reinforcing the correlation between implant survival and primary stability by increasing the bone-implant contact surface-an aspect in which wide-thread implants may play a crucial role. In the same line, Attia (19) evaluated immediate implants with different prosthetic connection configurations-platform switching (PS) versus platform matching (PM)-and observed comparable survival rates in both groups, even with follow-ups extending up to 23 years. These findings further support the long-term reliability of the immediate placement protocol. Taken together, although available studies on the survival of immediately placed implants with wide-thread macrogeometry remain scarce and show methodological limitations (small sample sizes and limited follow-up periods), the results consistently support the clinical viability of this protocol, provided that adequate primary stability is achieved. These findings form the basis for comparing the performance of immediate versus delayed placement, which will be addressed in the following section. 2. Comparison Between Immediate and Delayed Placement The choice between immediate and delayed implant placement remains a subject of clinical debate. While immediate placement offers advantages such as reduced treatment times, fewer surgical interventions, and preservation of alveolar bone, delayed placement has traditionally been considered more predictable in terms of osseointegration. This section reviews comparative studies that analyze survival rates, a key factor in determining the most appropriate protocol. Chatzopoulos and Wolff (20) conducted a large-scale, multicenter retrospective analysis aimed at evaluating and comparing survival rates of implants placed immediately and those placed after a delayed protocol. Records from 10 U.S. university dental clinics were analyzed, comprising a total of 50,333 implants in 20,842 patients between 2011 and 2022. Most patients (86.9%) received delayed implants, while 13.1% underwent immediate placement. Delayed implants showed a survival rate of 98.6%, compared to 98.4% for immediate implants, with no statistically significant difference between groups. These findings reinforce the clinical evidence supporting the viability of immediate placement, achieving outcomes comparable to delayed placement. This notion is further supported by a systematic review and meta-analysis conducted by Patel (21), which focused on the survival of immediate versus delayed implants. The review included 10 studies published between 2014 and 2022, comprising six randomized controlled trials and four non-randomized comparative studies. A total of 341 immediately placed implants were analyzed, of which 332 survived (97.4%), compared with 359 delayed implants, of which 350 survived (97.5%). The meta-analysis demonstrated no significant difference in survival rates between groups (risk ratio 0.99; 95% CI: 0.96-1.02; Z = 0.75; p = 0.45), confirming that immediate placement can be a predictable option provided adequate primary stability is achieved. Taken together, the available scientific evidence indicates that survival rates of immediate implants are comparable to those of delayed implants, supporting the clinical viability, reliability, and predictability of immediate protocols when sufficient primary stability is obtained. As primary stability depends largely on implant macrogeometry and thread design, the choice of configurations that optimize initial bone contact is critical to achieving outcomes comparable to delayed protocols. In addition, Peitsinis (22) conducted a literature review comparing clinical outcomes of immediate, early, and delayed implant placement. Randomized controlled trials, prospective studies, and retrospective studies were included, with implant survival as the primary outcome. Results showed high survival rates for immediate placement (93.8-100%), similar to early placement (4-16 weeks post-extraction) (95-100%) and delayed placement (4 months) (92-100%). The author emphasized that, in order to avoid complications such as marginal bone loss (MBL) under immediate protocols, treatment decisions should be individualized according to anatomical, esthetic, and patient-related factors-contexts in which thread macrogeometry plays a key role. Nevertheless, the results reinforce that immediate placement can achieve predictable outcomes in favorable clinical conditions. Further evidence comes from Grognard (23), who conducted a retrospective clinical study involving 63 patients (42 women and 21 men) treated with variable-thread implants (Nobel Active) featuring two platform configurations: regular platform (RP, Ø 4.3 mm) and narrow platform (NP, Ø 3.5 mm), all placed in the maxilla. Two protocols were compared: late implant placement (LIP, 2-3 months of healing; 42 implants) and immediate placement (IMIP, 6-8 months of follow-up; 34 implants). The primary outcome was secondary stability, assessed through ISQ values. Results showed that, for NP implants, mean ISQ values (±SD) were 70.84 (±4.86) in the LIP group and 72.41 (±3.89) in the IMIP group. For RP implants, mean ISQ values were 73.45 (±8.77) in LIP and 75.93 (±5.73) in IMIP. Immediate implants, therefore, achieved slightly higher ISQ values compared to delayed implants, although the difference was not statistically significant. The author concluded that immediate placement of variable-thread implants in the maxilla is clinically viable, achieving stability levels comparable to delayed placement, underscoring the importance of thread design in the predictability of immediate protocols. However, the study revealed an inconsistency in patient cohort reporting: the abstract indicated 63 patients (42 women, 21 men), whereas the methodology section reported 63 patients (52 women, 24 men). Although this discrepancy does not invalidate the main findings, it highlights the need for cautious interpretation and underscores certain limitations in methodological transparency. Overall, the available evidence suggests that variable-thread implants may provide advantages in implant-bone interface stability under immediate protocols, a scenario where wide-thread designs may add further value. Nevertheless, methodological inconsistencies in some studies and the lack of randomized controlled trials limit the strength of these conclusions. Well-designed prospective studies with transparent cohort descriptions and longer follow-ups are needed to confirm whether these biomechanical advantages translate into higher long-term clinical survival.

## Marginal Bone Loss (MBL): Medium- and Long-Term Behavior

Marginal bone loss (MBL) is one of the fundamental clinical and radiographic parameters for evaluating the long-term stability of implant treatment. This metric reflects the behavior and response of crestal bone to different surgical conditions and prosthetic loading protocols. The anatomical design of the implant-particularly thread configuration and the incorporation of strategies such as platform switching (PS)-directly influences bone preservation and peri-implant health. This section reviews the most relevant clinical and experimental evidence on the relationship between implant macrogeometry and long-term marginal bone stability. Several clinical studies have reported that the use of tapered implants with aggressive thread design promotes greater primary stability and, consequently, may improve bone preservation. In the randomized clinical trial by Messias (24), self-tapping tapered Screw-Line implants (Camlog®) with PS were compared with platform matching (PM). Both groups received the corresponding prosthetic components and were followed for five years. In the PM group, 33 patients received 72 implants, while in the PS group, 35 patients received 74 implants. The overall survival rate of implants reaching the final follow-up was 96.6%. From prosthetic loading to the 60-month evaluation, the PS group exhibited a bone gain of 0.19 ± 0.53 mm, whereas the PM group showed residual bone loss of 0.04 ± 0.58 mm, yielding a statistically significant mean difference of 0.23 mm (95% CI [0.03-0.43], p = 0.025). From surgery to 60 months, the mean difference increased to 0.34 mm (95% CI [0.14-0.54], p = 0.001). The authors concluded that PS may be beneficial for maintaining cervical bone in the long term. Although the study's main focus was the interface design (PS vs. PM), the evaluated implant (tapered, self-tapping, with aggressive threads) was conceived to favor primary stability through enhanced bone compression-a principle shared with wide-thread implants. This suggests that the bone-preserving benefits observed with PS could be further amplified when combined with macrogeometries that optimize initial bone contact, such as wide-thread designs. Additionally, Aung (18) assessed MBL in a retrospective clinical study previously discussed, including 49 tapered self-tapping Luna® implants placed immediately in 34 patients (23 mandibular, 26 maxillary). The mean follow-up period was 7.43 years (range 5-14 years). MBL was measured radiographically at different intervals (immediately post-surgery, at 3 months, 1 year, and 5 years), using a standardized digital calibration system. Results showed significant mesial bone gain from 3 months after placement (0.68 ± 1.61 mm), and progressive bone gain in both mesial and distal surfaces at 1 year, which remained stable up to 5 years compared to baseline (p &lt; 0.05). The author concluded that tapered self-tapping implants allow for stable long-term bone outcomes in immediate protocols, reinforcing the link between primary stability and cervical bone preservation. This constitutes valuable clinical evidence supporting marginal bone maintenance in immediate implant placement, consolidating its viability as a long-term therapeutic strategy. In a retrospective cohort study, Alqhtani (25) analyzed 180 implants in 120 patients divided into two groups (90 short implants 8 mm and 90 conventional implants 10 mm). Over a 5-year follow-up, success rates were 92.5% for short implants and 95.6% for conventional implants (p = 0.42). MBL analysis revealed means of 1.0 ± 0.5 mm for conventional implants and 1.3 ± 0.6 mm for short implants, with no statistically significant difference (p = 0.15). Probing depths (PD) were also comparable, with mean PD at 5 years of 2.6 ± 0.4 mm (short) and 2.4 ± 0.3 mm (conventional) (p = 0.38). These findings highlight that primary stability and bone quality may play a more decisive role in long-term maintenance than implant length itself. Importantly, many short implant designs incorporate wide or deep threads to optimize bone contact and compensate for reduced length, exemplifying the clinical relevance of macrogeometry in marginal bone preservation. In another retrospective clinical study, Attia (19) included 37 patients divided into PS (21 patients) and PM (16 patients), with follow-ups ranging from six months to 23 years. All patients received immediate endosseous implants after tooth extraction at the University Hospital of Giessen, Germany, between 2000 and 2023. Among them, 57% received PS implants and 43% PM implants. The most commonly used system was BEGO® RI (tapered, active-thread design) (83.8%), with 4.50 mm diameter implants being most frequent (56.8%), along with lengths of 13 mm (40.5%) and 15 mm (37.8%). Mean mesial bone loss was 0.26 mm in the PS group versus 0.75 mm in the PM group, while distal bone loss was 0.68 mm (PS) and 0.53 mm (PM). A statistically significant difference was observed mesially, favoring PS (p = 0.044). Larger implant diameters tended to reduce mesial bone loss and also distal bone loss, though not significantly. The authors concluded that while survival was comparable between groups, PS conferred an additional advantage in cervical bone preservation. Again, although the study focused on interface design, its immediate placement context reinforces the need to integrate configurations that favor both bone preservation (PS) and primary stability, where wide-thread designs can play a complementary role. One of the most methodologically robust investigations on MBL and thread design is the non-randomized, double-blind retrospective clinical study by Ormianer (2). A total of 1,361 implants were analyzed with a mean follow-up of 107 months (8.9 years). Three Alpha-Biotech® implant types with different thread designs were compared: Group A (SPI implants): tapered body and core, double-lead progressive thread with wide pitch; coronal threads accentuated toward the mid-apical portion, with narrow V-shaped apical core-considered wide and deep thread implants. Group B (DFI implants): also double-thread progressive design, but with smaller pitch and shallower apical V-threads, thus representing a more conventional thread design. Group C (Arrow implants): narrow, one-piece implants with a single V-thread, tapered body and core, and rounded narrow apex. Patients in Group A received 388 implants, those in Group B 911 implants, and those in Group C 62 implants. Of the 1,361 implants, 50 failed, yielding an overall survival rate of 96.3%. Mean MBL was 2.02 ± 1.70 mm (Group A), 2.10 ± 1.73 mm (Group B), and 1.90 ± 1.40 mm (Group C). Group A (wide, deep threads) showed significantly less bone loss than Group B (p = 0.036). These results confirm that wide and deep thread macrogeometry contributes to superior long-term crestal bone preservation compared with conventional designs, even when other body and insertion parameters are similar. The large sample size and long follow-up reinforce the strength of this evidence, positioning the study as a key reference supporting the utility of wide-thread implants in marginal bone stability. Importantly, this provides direct clinical evidence that beyond interface strategies such as PS, wide threads not only optimize primary stability but also translate into improved medium- and long-term marginal bone maintenance. Table 2 presents marginal bone loss values according to thread design from clinical and retrospective studies.

Table 2

##  Critical Discussion

1. Strengths of Wide-Thread Implants in Immediate Situations Wide-thread implants have demonstrated significant advantages in clinical scenarios involving immediate placement and early loading protocols. First, they stand out for their ability to achieve high levels of primary stability, even under low bone density conditions. Studies such as those by Cucinelli (1) in vitro and Baldi (10) in patients have shown that greater thread depth and thickness translate into higher insertion torque and superior ISQ values-critical parameters for success in immediate protocols. From a biomechanical perspective, the literature supports that wide-thread designs promote a more favorable distribution of stresses within the bone, reducing load concentration in critical areas. Three-dimensional analyses, such as that of Alqahtani AR (7), indicate that square-thread configurations with reduced pitch optimize load transmission, thereby reinforcing mechanical predictability in compromised bone scenarios. At the clinical level, encouraging medium-term outcomes have been reported. Ormianer (2) documented lower MBL in wide-thread implants compared with conventional designs, while Coppedê (8) confirmed that All-on-Four protocols with high insertion torque values (75 Ncm) and stability monitored through RFA remained clinically safe over follow-ups of up to 24 months. These findings suggest that wide threads provide not only immediate benefits but also contribute to maintaining functional implant stability over time. In line with these results, Aung (18) reported high cumulative survival rates and stable marginal bone maintenance after prolonged mean follow-up in immediately placed, self-tapping tapered implants, further reinforcing the reliability of such designs in long-term immediate protocols. Finally, the adaptability of wide-thread implants proves particularly useful in challenging situations such as post-extraction placement or in trabecular bone of low density, where the combination of more aggressive macrogeometries with tapered, self-tapping apices favors effective anchorage and reduces the risk of micromovement during the early phases of osseointegration. Taken together, the available evidence supports wide-thread implants as an effective tool to improve predictability in immediate and early loading protocols, offering superior primary stability, biomechanical advantages, and clinical outcomes that-although still requiring long-term validation-consolidate their role as a preferred option in highly demanding clinical scenarios. 2. Limitations of the Available Studies While both experimental and clinical evidence support the role of wide-thread macrogeometry in enhancing primary stability and predictability under immediate protocols, it is important to acknowledge the inherent limitations of the available studies. One of the main weaknesses lies in methodological heterogeneity: different investigations rely on in vitro models, finite element analysis (FEA) simulations, or retrospective clinical cohorts, making it difficult to establish direct comparisons and uniformly translate findings into clinical practice. In addition, the included literature comprised heterogeneous types of evidence, including clinical studies, in vitro experiments, and finite element analyses. To minimize the risk of overinterpreting experimental findings, the available evidence was interpreted according to a hierarchical framework in which clinical studies were considered the primary source of information for evaluating clinical outcomes, while experimental and simulation-based studies were used to provide mechanistic support. In addition, certain inconsistencies raise concerns about the robustness of the reported conclusions. For instance, in the FEA study by Herekar (9), although the results demonstrated improved performance of multiple-microthread designs, the author concluded that a single-microthread configuration would be more favorable overall-an interpretation that contradicts the presented findings. Similarly, in the retrospective clinical study by Grognard (23), a discrepancy was noted in the description of the patient cohort: while the abstract reported 63 patients (42 women and 21 men), the methodology section described 63 patients (52 women and 24 men). Although these divergences do not invalidate the main outcomes, they highlight deficiencies in methodological transparency and emphasize the need for cautious data interpretation. Another recurrent limitation is the absence of randomized controlled trials (RCTs) with extended follow-up. Most available studies are characterized by small sample sizes, short observation periods (5 years), and a lack of standardization in the measurement of critical variables such as insertion torque or ISQ values. Together, these shortcomings hinder the establishment of strong correlations between wide-thread design and long-term clinical survival. Within this context, although current findings appear promising, they must be interpreted with caution, recognizing that the strength of the evidence is restricted by methodological inconsistencies, modest sample sizes, and the absence of robust long-term clinical validation. These observations are consistent with previous reviews on implant macrogeometry, while adding a specific focus on wide-thread designs under immediate placement protocols. This review also presents methodological limitations, including the absence of prospective protocol registration, the use of a single primary database supplemented by manual screening, and the use of a single reviewer for screening and data extraction. These factors may introduce potential selection bias and should be considered when interpreting the findings. 3. Proposals for Future Studies The current evidence on wide-thread implants in immediate placement contexts, while promising, is limited by methodological constraints that hinder the extrapolation of findings to routine clinical practice. In this regard, several priority research directions can be identified: 1. Multicenter randomized controlled trials (RCTs). Well-designed controlled trials with large sample sizes are needed to compare wide-thread implants against conventional designs, both under immediate and delayed protocols, in order to establish consistent differences in terms of primary stability, marginal bone loss, and long-term survival. 2. In vivo biomechanical analyses. Although finite element analysis (FEA) studies provide valuable insights, they should be complemented with dynamic clinical records of insertion torque, ISQ values, and micromovements under functional load. This approach would allow validation of the real-world applicability of experimental models and strengthen the correlation between mechanical parameters and clinical outcomes. 3. Long-term follow-up studies. Most available data focus on short- and medium-term outcomes (5 years). Further research with follow-up periods exceeding 5 years is required to determine whether the advantages observed in primary stability and load biomechanics translate into higher long-term survival and stable peri-implant bone preservation. Taken together, progress in these areas would not only confirm the clinical value of wide-thread implants in immediate placement scenarios but also help to define more precisely their indications and the limits of their applicability. Ultimately, although the strengths of wide-thread implants in immediate protocols are evident, interpretation of the results must be tempered by the methodological limitations of the current literature. Only through well-designed clinical studies will it be possible to confirm their true impact on long-term survival.

## Conclusions

Within this systematic review, the available evidence supports wide-thread implants as an effective strategy to enhance primary stability in demanding clinical scenarios such as immediate post-extraction placement and early or immediate loading protocols. Experimental, in vitro, and finite element analyses consistently indicate that their macrogeometry promotes greater mechanical retention, more favorable stress distribution, and higher insertion torque and ISQ values compared with conventional thread configurations. At the clinical level, short- and medium-term outcomes are encouraging, demonstrating high success rates and a trend toward reduced marginal bone loss (MBL). Nevertheless, the methodological heterogeneity of available studies-characterized by the absence of standardized key parameters, inconsistencies in some reports, and the lack of randomized controlled trials with extended follow-ups-limits the robustness of current evidence. Accordingly, while wide-thread implants appear to be a valuable tool for improving the predictability of immediate protocols, confirmation through well-designed multicenter studies with standardized methodologies and long-term studies is still required to determine whether these early advantages translate into superior long-term clinical survival and stable bone preservation.

## Figures and Tables

**Table 1:** Insertion torque (IT) and ISQ according to thread design: synthesis of experimental and clinical studies.

Study (Author, year)	Insertion torque (IT, Ncm)	ISQ	Key notes
Baldi (2018)	18.8–68.2	71.8–78	Clinical; positive IT–ISQ correlation
Shiigai (2007)	49 (in vitro); 43.2 (clinical)	70 (in vitro); 71.8 (clinical)	Triple thread improved IT and ISQ
Cucinelli (2024)	54.03 ± 8.99 (deep) vs 25.12 ± 2.96 (shallow)	70.13 ± 1.12 (deep) vs 65.58 ± 1.37 (shallow)	Deeper threads increased IT and ISQ in D3 bone
Ayub (2025)	30.21 ± 1.38 (Zimmer, D3); 18.07 ± 1.71 (Zimmer, D4)	63.29 ± 0.54 (Dentium, D3); 58.46 ± 0.78 (Dentium, D4)	Conical implants with deep/aggressive threads more stable in D3/D4
Yamaguchi (2020)	NR	Improved vs control (NR)	Double/single thread; length and pitch influenced stability
Menini (2020)	48.42 (SL) vs 43.42 (SY)	NR	Clinical; SL (wide thread) showed higher IT than SY
Huang (2023)	NR	NR	Review; need to control micromovements (<0.1 mm)
Lozano-Carrascal (2016)	46.67 ± 6.85 mandibular (conical) vs 35.77 ± 6.72 mandibular (cylindrical)	71.67 ± 5.16 mandibular (conical) vs 57.15 ± 4.83 mandibular (cylindrical)	Conical more favorable in mandible and maxilla; significant difference
Ryu (2014)	NR	NR	Review; aggressive threads recommended in compromised bone
Oliveira (2016)	≥ 30 Ncm recommended	NR	Review; ≥30 Ncm associated with >95% success
Coppedê (2025)	≥ 75	Stable at 2 years (NR)	All-on-Four; high IT and stable ISQ at 2 years

1

**Table 2:** Marginal bone loss (MBL) according to thread design: synthesis of clinical and retrospective studies.

Study (Author, year)	Design / Comparison	Sample and follow-up	Key results (MBL, mm)
Messias (2019)	Screw-Line (Camlog®), PS vs PM	74 PS / 72 PM; 5 years	PS: gain 0.19 ± 0.53 mm vs PM: loss 0.04 ± 0.58 mm; mean difference 0.23 mm ( p = 0.025)
Aung (2024)	Luna®, Tapered, self-tapping	34 patients; 49 implants; 7.4 years	Mesial and distal bone gain maintained up to 5 years (p <0.05); survival rate 93,9%
Alqhtani (2024)	Short implants (≤ 8 mm) vs conventional (≥ 10 mm)	120 patients; 180 implants; 5 years	Mean MBL: 1.0 ± 0.5 mm (conventional) vs 1.3 ± 0.6 mm (short); NS ( p = 0.15)
Attia (2025)	BEGO® RI, PS vs PM	37 patients; 6 months–23 years	Mesial loss: PS 0.26 mm vs PM 0.75 mm (p = 0.044). Distal not significant
Ormianer (2016)	Alpha-BioTech® SPI (wide and deep thread) vs DFI (standard) vs Arrow	1361 implants; 8.9 years	MBL: SPI 2.02 ± 1.70 mm vs DFI 2.10 ± 1.73 mm vs Arrow 1.90 ± 1.40 mm; SPI lower than DFI ( p = 0.036)

2

## Data Availability

The datasets used and/or analyzed during the current study are available from the corresponding author.
